# A Smart Health Platform for Measuring Health and Well-Being Improvement in People With Dementia and Their Informal Caregivers: Usability Study

**DOI:** 10.2196/15600

**Published:** 2020-07-23

**Authors:** Estefania Guisado-Fernandez, Catherine Blake, Laura Mackey, Paula Alexandra Silva, Dermot Power, Diarmuid O'Shea, Brian Caulfield

**Affiliations:** 1 Insight Centre for Data Analytics Dublin Ireland; 2 School of Public Health, Physiotherapy and Sports Science University College Dublin Dublin Ireland; 3 Department of Informatics Engineering (DEI) Center for Informatics and Systems of the University of Coimbra (CISUC) University of Coimbra Coimbra Portugal; 4 Medicine for the Elderly Mater University Hospital Dublin Ireland; 5 Department of Geriatric Medicine Saint Vincent's University Hospital Dublin Ireland

**Keywords:** Connected Health, dementia, informal caregiver, home care, home monitoring

## Abstract

**Background:**

Dementia is a neurodegenerative chronic condition characterized by a progressive decline in a person’s memory, thinking, learning skills, and the ability to perform activities of daily living. Previous research has indicated that there are many types of technology interventions available in the literature that have shown promising results in improving disease progression, disease management, and the well-being of people with dementia (PwD) and their informal caregiver, thus facilitating dementia care and living. Technology-driven home care interventions, such as Connected Health (CH), could offer a convenient and low-cost alternative to traditional home care, providing an informal caregiver with the support they may need at home while caring for a PwD, improving their physical and mental well-being.

**Objective:**

This study aimed (1) to create a multidimensional profile for evaluating the well-being progression of the PwD–informal caregiver dyad for a year during their use of a CH platform, designed for monitoring PwD and supporting their informal caregivers at home, and (2) to conduct a long-term follow-up using the proposed well-being profile at different time-interval evaluations.

**Methods:**

The PwD–informal caregiver well-being profile was created based on the World Health Organization International Classification of Functioning considering the following outcomes: functional status, cognitive status, and quality of life for the PwD and mental well-being, sleeping quality, and burden for the informal caregiver. Over a year, comprehensive assessments of these outcomes were conducted every 3 months to evaluate the well-being of PwD–informal caregivers, using international and standardized validated questionnaires. Participants’ demographic information was analyzed using descriptive statistics and presented as means and SDs. A nonparametric Friedman test was used to analyze the outcome changes and the progression in the PwD-caregiver dyads and to determine if those changes were statistically significant.

**Results:**

There were no significant changes in the well-being of PwD or their caregivers over the year of follow-up, with the majority of the PwD-caregiver dyads remaining stable. The only instances in which significant changes were observed were the functional status in the PwD and sleep quality in their caregivers. In each of these measures, post hoc pairwise comparisons did not indicate that the changes observed were related to the deployment of the CH platform.

**Conclusions:**

The follow-up of this population of PwD and their informal caregivers has shown that disease progression and physical and mental well-being do not change significantly during the time, being a slow and gradual process. The well-being profile created to analyze the potential impact of the CH platform on the PwD–informal caregiver dyad well-being, once validated, could be used as a future tool to conduct the same analyses with other CH technologies for this population.

**International Registered Report Identifier (IRRID):**

RR2-10.2196/13280

## Introduction

### Background

Dementia is a neurodegenerative chronic condition characterized by a progressive decline in a person’s memory, thinking, learning skills, and the ability to perform activities of daily living (ADLs) [1]. Currently, dementia affects 47 million people worldwide, and these numbers are expected to increase to 75 million by 2030 and 132 million by 2050 [2]. A diagnosis of dementia has a significant impact on family members of people with dementia (PwD), who often bear the responsibility of caring for them as they deteriorate [3]. These family members, usually a spouse or a child, are often referred to as *informal caregivers*, as they offer continuous unpaid assistance, in contrast to *formal caregivers*, who offer paid professional services [3]. Informal caregiving can help to maintain the PwD at home, avoiding institutionalization and providing the *Aging in Place* model of care; avoiding nursing home placement; and contributing to an increase in well-being, independence, social participation, and healthy aging [4].

Previous research has indicated that there are many types of technology interventions available in the literature that have shown promising results in improving disease progression, disease management, and the well-being of PwD and their informal caregivers, thus facilitating dementia care and living [5,6]. This is the case for Connected Health (CH), a model of chronic care delivery facilitated by technology where all the stakeholders involved in a person’s care are *connected* through a health portal that provides a continuous and efficient flow of information between them [7]. The concept of CH has gained attention among dementia researchers, as it has shown positive results in helping informal caregivers in their delivery of home care for the elderly [8,9]. Using a wide variety of technologies such as body-worn and monitoring devices, CH can help the informal caregiver in their caring duties through the continuous monitoring of the health status of the PwD at home, alerting them to changes in the PwD and their environment (such as falls or any other emergency event), and facilitating communication with health care professionals (HCP) when needed. CH-driven interventions could offer a convenient and low-cost alternative to traditional home care, providing an informal caregiver with reliable information and social and emotional support as well as enhancing information exchange with other caregivers and HCPs, facilitating the informal caregiver the decision-making process for matters concerned with PwD care [10]. The literature also suggests that many of these types of technology-driven interventions are designed to provide well-being to informal caregivers, helping to ameliorate the levels of burden and stress they can feel derived from their caring process [11]. Similarly, technology applied for dementia home care might play a role in PwD monitoring and disease decline prevention through the detection of changes in the PwD ADLs performance or physical parameters, alerting the caregiver and the HCP to act in advance and prevent further complications (eg, falls prevention, disease relapse, or hospitalization) [11]. At the same time, these technologies aim to empower the informal caregiver and increase their confidence and self-efficacy in their care role, improving the quality of life (QoL) and well-being of PwD and their informal caregivers [10]. An excellent example of a combination of patient home monitoring and informal caregiver support is the ALADDIN project, conducted by Torkamani et al. in 2014 [12]. ALADDIN is a digital platform designed to offer support to the informal caregiver through the provision of information (*Television* and *Social Networking*), a communication tool with formal carers (*Contact us*), and a distant monitoring feature (*My tasks*) where the informal caregiver had to complete a questionnaire that gathered information about the PwD health. It was tested in a multisite randomized controlled pilot study with 30 community living informal caregivers of PwD. The intervention and control groups were assessed at baseline, at 3 months, and at 6 months in terms of burden depression and QoL for the caregiver and for cognitive and disease stage, functional disability, comorbidities, and QoL for the PwD. The authors reported a significant improvement in the QoL of the carers in the platform group, with some reduction in caregiver burden and distress, and that the platform was useful in monitoring the PwD and facilitating contact with other professionals. In addition, caregivers and clinicians rated the access to and use of the ALADDIN platform positively. The success of studies such as this supports further testing of the utility and the value of technology interventions in other dementia cohorts, but they need to be studied for more extended periods to investigate the true impact that it can have on the PwD care. Furthermore, the addition of technology devices and wearables to monitor the vital signs of PwD can be a facilitator in this remotely telemonitoring process.

On the basis of the literature knowledge, this study aimed to create a well-being profile of the PwD–informal caregiver dyads involved in *Connected HEalth Sustaining home Stay* (CHESS) in dementia project, a CH study, to help to report their progression during their year of involvement in the study to see if there was any impact on it because of the use of a CH platform for home care.

### CHESS Project Overview

**Figure 1:**
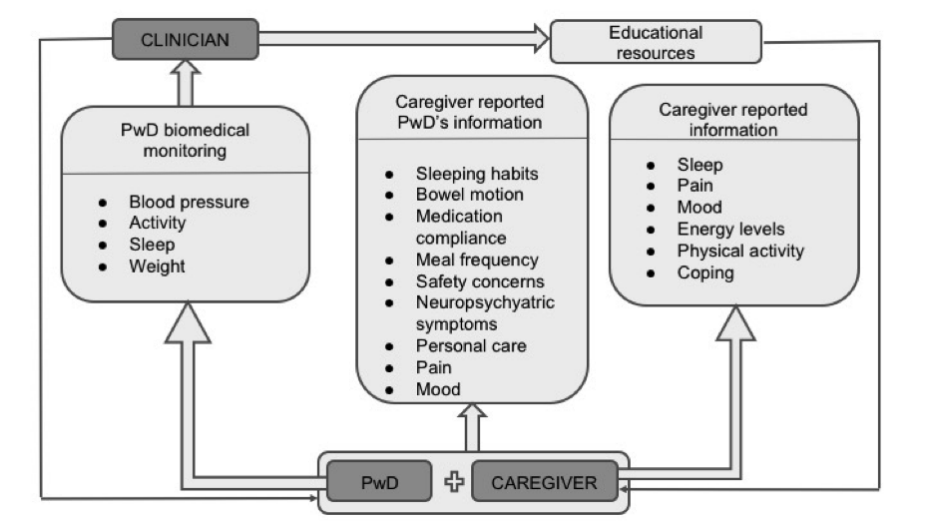
Representation of the Connected Health platform components.

CHESS is a CH longitudinal cohort study that took place in the University College Dublin (UCD, Ireland) between the beginning of 2016 and the end of 2019. The project aimed to (1) evaluate the effectiveness of a CH platform in supporting informal caregivers of PwD at home, compared with usual care; (2) study the impact of CH on dementia home care, in terms of the potential improvement of the PwD and their informal caregivers’ physical and mental health and QoL; and (3) to determine the CH platform’s usability and user experience from the informal caregivers’ perspectives. The full CHESS project protocol has already been published [13]. The CH platform works on a tablet computer (Samsung Galaxy Tab A 10.1, 2016) and is connected to a series of PwD monitoring devices for home use, including a blood pressure (BP) monitor (Omron M6 by OMRON Healthcare Ltd), an electronic weighing scale (Withings, France), and an activity and sleeping tracker (Withings Go). The platform provides 4 features to the informal caregivers: an educational section with information and videos from dementia experts offering advice about daily care; an assessment module with daily questionnaires for the informal caregivers that collects health-related information about themselves and the PwD (in the case of PwD, data on their mood, nutrition, activity, bowel movements, and medication compliance are collected; for caregivers, surveys on their mood, energy levels, sleep quality, and anxiety levels are conducted); a diary for the caregivers to keep track of events, with summary reports of changes in the PwD care plan; and a dashboard with an overview of the PwD activity levels, sleep patterns, BP, and weight, recorded by the monitoring devices. The encrypted platform securely connects all the key stakeholders involved in PwD’s care (ie, informal caregiver, general practitioner, public health nurse, and hospital geriatric services). As mentioned earlier, the generated data are presented on the platform and made available for the informal caregivers and HCPs as an objective measure of the PwD’s health status. Figure 1 shows a representation of the CH platform components. Screenshots of the platform interface, sections, and devices can be found in Multimedia Appendix 1.

A preliminary subjective feedback study was conducted from a sample of our participants. This preliminary study showed that their initial impressions about what the CHESS platform could offer to them to improve their delivery of home care for the PwD did not correspond with what they found. In the beginning, they considered the platform as a tool to enhance their caring tasks and to improve their self-efficacy. After the deployment, they considered the platform to be more helpful for research than for themselves. This study has already been published, and more information about these informal caregivers’ subjective experience can be found in the manuscript [14].

## Methods

### Study Aims

In this study, we aimed (1) to create a multidimensional profile for evaluating the progression of the well-being of PwD–informal caregiver dyads during their use of the CH platform and their involvement in the CHESS study (1 year) and (2) to conduct a long-term follow-up using the proposed well-being profile, including different time-interval evaluations.

### Study Design

This study reported a longitudinal quantitative analysis of the well-being of PwD-caregiver dyads that were involved in the *CHESS* project during a year’s time, using a well-being profile created for the occasion.

#### Participants’ Well-Being Profile Creation and Assessment

**Figure 2:**
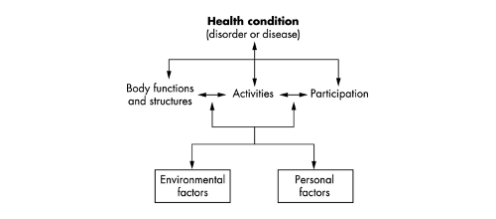
World Health Organization International Classification of Functioning framework.

The PwD–informal caregiver well-being profile was created based on the World Health Organization (WHO) International Classification of Functioning (ICF) [15]. The ICF is a framework for describing and organizing an individual’s information about functioning, health, and disability (Figure 2). In it is reflected how a disease or a health condition can make an impact on an individual in 3 main domains: body functions and structures, activities, and social participation. This impact may lead to a restriction in socialization and isolation and, therefore, affect well-being. These domains encompass all the physical, mental, and social aspects that define a person’s well-being. As clinicians, we have to consider a person or a patient as a whole entity. Therefore, a person’s well-being cannot be defined just by one of the following domains: their physical and mental functioning or social relationships. Our aim was to use this framework to create a PwD–informal caregiver well-being profile that comprehends all those aspects of a person’s well-being and to use this as a tool for evaluating their well-being progression during the year they were involved in the study.

Applying this framework to the PwD, we created a well-being profile considering the PwD’s following outcomes:

- The PwD functional status (body functions and structures domain): as a measure of disability
- The PwD cognitive status (activities domain): as a measure of their limitation in performing ADLs
- The PwD QoL (participation domain): as a measure of their social participation restriction.

For the informal caregiver, we created a well-being profile considering the following outcomes:

- The informal caregiver’s mental health wellness, including anxiety, depression, and stress (body functions and structures domain): as a measure of the impact that their mental well-being can have on their body functions and how they respond to the daily caring demands
- The informal caregiver sleep quality (activity domain): as a measure of the impact that the lack of sleep can have in performing their daily caring tasks
- The informal caregiver burden (participation domain): as a measure of their social participation restriction.

These outcomes were evaluated using a series of validated international questionnaires:

- For the PwD:
  PwD-related functional status was evaluated with the help of the Disability Assessment Dementia (DAD) scale [16]. The DAD scale was initially designed for community-based individuals with Alzheimer dementia, but it has been recently used in other types of dementia research. It is a tool used by the HCP to investigate the PwD levels of dependency and to guide the provision of tailored interventions for PwD. In addition, as a research tool, it can be used to describe the functional characteristics of PwD and the progression of the disease. A total score is converted out of 100, with the result of a percentage that provides an understanding of the PwD global function in ADLs. Higher scores indicate less disability in conducting ADLs, with lower scores indicating more dysfunction and more dependency on the caregiver [16].
  PwD cognitive status was measured using the Mini-Mental State Examination (MMSE) [17,18]. The MMSE is composed of 11 questions that cover 5 areas of cognitive function: orientation, registration, attention and calculation, recall, and language. The maximum score was 30, with a score of 23 or less being indicative of cognitive impairment. This is a quick and easy tool to administer directly with the PwD and is very useful when conducting it repetitively [17,18].
  PwD QoL was measured using the self-reported Dementia Quality of Life (DEMQoL) scale [19,20]. DEMQoL is designed to work across dementia subtypes and care arrangements and is suitable for all stages of the disease. It comprised 2 questionnaires: (1) DEMQoL: a 28-item questionnaire answered by the PwD (self-reported QoL), and (2) DEMQoL-Proxy: a 31-item questionnaire answered by the caregiver (PwD’s caregiver-reported QoL). Scored items are summed to produce a total score, with higher scores indicating better health-related QoL [19,20].
- For the informal caregiver:
  The Hospital Anxiety and Depression Scale (HADS) was used to measure anxiety and depression levels [21,22]. The HADS is a brief and straightforward self-report questionnaire. A total summary score classifies the respondent into 3 groups, depending on their levels of depression or anxiety: normal, borderline case, or abnormal. This questionnaire does not provide a diagnosis, as it was created for screening purposes only [21,22].
  Caregivers’ sleep quality was determined using the Pittsburgh Sleep Quality Index (PSQI) [23,24]. The PSQI is designed to evaluate the overall sleep quality for 1 month. It is a 19-item self-reported questionnaire with 7 subcategories: subjective sleep quality, sleep latency, sleep duration, habitual sleep efficiency, sleep disturbances, use of sleeping medication, and daytime dysfunction. This questionnaire was initially created to measure the sleep quality in psychiatric populations but has been widely used for clinical and research purposes [23,24].
  Caregiver burden was evaluated using the Zarit Burden Interview (ZBI) scale [25,26]. It is comprised of 22 questions about the impact of the PwD’s disabilities on caregivers’ lives and has been designed to reveal the stress experienced by the caregiver. For each item, the caregivers must indicate how burdened they are (never, rarely, sometimes, quite frequently, or nearly always). A total score can be calculated from the summing of each answer, with higher scores indicating higher levels of burden and stress due to the caring process [25,26].

Table 1 provides details on the PwD–informal caregiver dyads well-being profile created based on the WHO ICF.

**Table 1:** People with dementia and informal caregiver well-being profile based on the World Health Organization International Classification of Functioning framework.

Domains	Person with dementia	Caregiver
Body structure and function	Mini-Mental State Exam (cognitive function)	Hospital Anxiety and Depression Scale (anxiety and depression) Zarit Burden Interview (stress/distress)
Activity limitation	Disability Assessment Dementia (functional status)	Pittsburgh Sleep Quality Index (sleep quality)
Participation restriction	Dementia Quality of Life and Dementia Quality of Life-proxy (quality of life)	Zarit Burden Interview (participation)

#### Timing of Measurements

During the year of follow-up, comprehensive assessments to evaluate the well-being of PwD–informal caregivers were conducted every 3 months following the study protocol [13], at 0, 3, 6, 9, and 12 months, using the international and standardized validated questionnaires described earlier. They were completed electronically on the researchers’ administrators’ interface of the platform by the caregiver and the patient, with the help of the researcher (see Table 2 for the comprehensive list of the well-being questionnaires and their timing during the 12-month follow-up).

**Table 2:** Quarterly comprehensive list of well-being evaluation questionnaires and timing with the informal caregiver and people with dementia during the year of follow-up.

Individuals	Month 0	Month 3	Month 6	Month	Month 12
**People with dementia**					
	DEMQoL^a^ MMSE^b^ DAD^c^ DEMQoL-proxy	DEMQoL MMSE DAD DEMQoL-proxy	DEMQoL MMSE DAD DEMQoL-proxy	DEMQoL MMSE DAD DEMQoL-proxy	DEMQoL MMSE DAD DEMQoL-proxy
**Caregiver**					
	HADS^d^ PSQI^e^ ZBI^f^	HADS PSQI ZBI	HADS PSQI ZBI	HADS PSQI ZBI	HADS PSQI ZBI

^a^DEMQoL: Dementia Quality of Life.

^b^MMSE: Mini-Mental State Examination.

^c^DAD: Disability Assessment Dementia.

^d^HADS: Hospital Anxiety and Depression Scale.

^e^PSQI: Pittsburgh Sleep Quality Index.

^f^ZBI: Zarit Burden Interview.

#### Study Participants

Our participants’ sample was recruited from the already participating dyads in the CHESS project. We included participants from June 2017 and who had completed the year follow up by December 2018. Ethical approval for this study was obtained, as part of the CHESS project, from the research ethics committees from the collaborating hospitals (Mater Misericordiae University Hospital and Saint Vincent’s University Hospital) and UCD Human Research Ethics Committee.

#### Statistical Analysis

Participants’ demographic information was analyzed using descriptive statistics and presented as means and SDs. Owing to the small sample size, the nonparametric Friedman test was used to analyze the changes in the outcomes and progression during the year of follow-up in the PwD (MMSE, DAD, DEMQoL, and DEMQoL-proxy) and their respective informal caregivers (HADS, PSQI, and ZBI) and to determine if those changes were statistically significant. In case of finding significant changes in any of the outcomes, post hoc pairwise comparisons analysis was conducted using the nonparametric Wilcoxon test to help understand specific differences between the different time intervals. Only some scales could be classified by ranges (MMSE, HADS, PSQI, and ZBI). Table 3 provide details of each variable’s scoring and classification. In the case of the scales in which scores were not classified by ranges (DAD, DEMQoL, and DEMQoL-proxy), only a description of the score changes was provided. Where data were missing, the analysis was based on the available data, without discarding any participant because of the small sample size recruited. All statistical data analyses were conducted using SPSS version 24 for Mac (IBM Corp, Released 2016; IBM SPSS Statistics for Macintosh, version 24.0).

**Table 3:** Mini-Mental State Examination score and range classification.

Scales and classifications		Scoring	
**Mini-Mental State Examination**			
	Mild cognitive impairment		25-30
	Mild dementia		21-24
	Moderate dementia		13-20
	Severe dementia		<12
**Hospital Anxiety and Depression Scale**			
	Normal		0-7
	Borderline		8-10
	Abnormal (case)		11-21
**Pittsburgh Sleep Quality Index**			
	Poor sleep		>5
	Normal		<5
**Zarit Burden Interview**			
	Little/no burden		0-21
	Mild/moderate		21-40
	Moderate/severe		41-60
	Severe		61-88

#### Minimal Clinical Significance Analysis

To further explore the participants’ changes and progression, their group and individual profiles were examined using minimum clinically significant changes in status. Minimal clinical significance has been established for several measures as follows:

- MMSE score at more than 3 points [27]
- DAD by 12 points [28]
- HADS by 1.5 points [29].

These cutoff points or thresholds were applied to identify clinically significant changes in our individuals during the year of follow-up. We did not find any cutoff points for DEMQoL, DEMQoL-proxy, PSQI, and ZBI scales in the literature. For these cases, we have just described the progression of our participants based on the score changes.

## Results

### Participant Characteristics

A total of 11 PwD–informal caregiver dyads were recruited. The informal caregivers had a reasonable balance between females and males (6/11, 54% female vs 5/11, 45% male), with an average age of 69.27 (SD 13.14) years. Most caregivers were spouses of the PwD (8/11, 72% cases), having been a dedicated caregiver for the PwD for an average of 3 (SD 2.69) years. Most of the informal caregivers were retired (8/11, 72% cases). In terms of the PwD they were caring for, there was a reasonable balance between genders (6/11, 54.5% female vs 5/11, 45.5% male), and the PwD had an average age of 75.09 (SD 10.13) years. The majority of the PwD had vascular dementia (4/11, 36%) or a nonspecified type of dementia (4/11, 36% cases). All the PwD were living at home with their informal caregivers. At enrollment time, the mean MMSE score was 24.10 (SD 3.66), indicating mild dementia, and the mean DAD score was 74.64 (SD 27.76). Tables 4 and 5 provide further details of the participants.

**Table 4:** Demographic characteristics of the people with dementia involved in the year of follow-up (N=11).

People with dementia demographics		Values
**Gender, n (%)**		
	Male	5 (45)
	Female	6 (54)
**Type of dementia, n (%)**		
	Vascular dementia	4 (36)
	Not specified	4 (36)
	Alzheimer disease	1 (9)
	Other (Parkinson disease)	1 (9)
	Lewy body	1 (9)
**Education, n (%)**		
	Primary	3 (27.3)
	Secondary	4 (36.4)
	Tertiary	2 (18.2)
	Postgraduate	2 (18.2)
**Mini-Mental State Examination** **levels at enrollment, n (%)**		
	Mild cognitive impairment	4 (36.36)
	Mild	4 (36.36)
	Moderate	3 (27.27)
	Severe	0 (0)
Disability Assessment Dementia score at enrollment, mean (SD)		74.64 (27.76)
Age, mean (SD)		75.09 (10.13)

**Table 5:** Demographic characteristics of the informal caregivers involved in the year of follow-up (N=11).

Caregiver’s Demographics		Values
**Gender, n (%)**		
	Male	5 (45)
	Female	6 (54)
**Caregiver-** **people with dementia** **relationship, n (%)**		
	Spouses	8 (72)
	Children	3 (27)
**Caregiver employment, n (%)**		
	Retired	8 (72)
	Part time	2 (18)
	Carers’ allowance	1 (9)
**Caregiver educational levels, n (%)**		
	Primary	2 (18)
	Secondary	4 (36)
	Tertiary	3 (27)
	Postgraduate	2 (18)
Caregiver age, mean (SD)		69.27 (13.14)
Caregiver years in care, mean (SD)		3.0 (2.69)

### PwD–Informal Caregiver Dyad Well-Being Progression During the Year of Follow-Up

#### PwD Well-Being Progression

From all of the outcomes analyzed, we only found significant changes in 2: the PwD functional status (DAD scale) and in the informal caregiver sleep quality (PSQI scale). When individual cases were analyzed, we found considerable variation between participants, reflected in changes on an individual basis for both the PwD and their informal caregivers. A detailed description of each outcome progression is described in the following sections.

The global cognitive function in our PwD population sample showed a small decrease in the mean MMSE of 1.5 (SD 0.9) points from baseline to the year of follow-up period. However, this did not reach the threshold for statistical significance at the 0.5 level (*P=*.61) in the Friedman test. The overall mean MMSE score was 23.25 (SD 4.77), indicating a mild dementia stage (4/11, 36% of participants). When looking at individual cases, 18% (2/11) of PwD decreased more than 3 points their MMSE score during the year of follow-up, experiencing a clinically significant cognitive decline. The other 9 PwD (81%) remained stable (changed by 3 points or less). See Tables 6 and 7 below for further details.

In terms of the functional status of our sample of PwD, the overall mean DAD score was 65.47 (SD 28.80), with a progressive deterioration during the year of follow-up, shown by a diminution of 11.39 points in the total DAD score. There was a significant difference across the 5 time points measurement during the year of follow-up (Friedman test *P=*.02), with a mean score at month 0 of 71.53 (SD 27.95) and a mean score at month 12 of 60.14 (SD 30.12).

When looking at individual cases, the DAD scores of 63% (7/11) of PwD dropped by more than 12 points during the year of follow-up, experiencing a clinically significant functional decline, whereas the other 4 PwD (36.36%) remained the same (changed <12 points). See Table 8 for more details.

**Table 6:** People with dementia Mini-Mental State Examination score during the year progression.

Mini-Mental State Examination	Month 0	Month 3	Month 6	Month 9	Month 12	Friedman test (*P* value)
Minimum	19	18	11	15	12	.06
Quartile 1	21.5	19	19	19.2	20	N/A^a^
Median	23.5	23	23	23.5	23.5	N/A
Mean (SD)	24.1 (3.6)	24.0 (5.0)	22.6 (5.9)	22.6 (4.3)	22.8 (5.0)	N/A
Quartile 3	27.2	29	27.5	24.7	26	N/A
Maximum	30	30	30	29	30	N/A

^a^N/A: not applicable.

**Table 7:** Number of people with dementia in each Mini-Mental State Examination range group at months 0 and 12.

Mini-Mental State Examination ranges	Mini-Mental State Examination Score	Participants at month 0 (n=11), n	Participants at month 12 (n=10), n
Mild cognitive impairment	25-30	4	4
Mild-Moderate	21-24	4	2
Moderate	13-20	3	3
Severe	<12	0	1

**Table 8:** People with dementia Disability Assessment Dementia score during the year progression.

Disability Assessment Dementia	Month 0	Month 3	Month 6	Month 9	Month 12	Friedman test (*P* value)
Minimum	27.7	20.5	15	12.5	12.5	.02
Quartile 1	49.7	50.5	39.4	27.5	38.1	N/A^a^
Median	80.2	80	71.7	60.5	65.1	N/A
Quartile 3	96.0	94.7	88.6	80	79.3	N/A
Maximum	97.4	100	100	100	100	N/A
Mean (SD)	71.5 (27.9)	72.1 (27.6)	65.1 (29.6)	58.4 (31.3)	60.1 (30.1)	N/A

^a^N/A: not applicable.

Post hoc pairwise comparisons analysis was conducted using a nonparametric Wilcoxon test to help understand specific differences between the different time intervals within the DAD results, to find where the significance difference relies on between the 5 time point measurements. The Wilcoxon test results revealed a statistically significant reduction in PwD DAD score from months 3 to 6, from months 6 to 9, from months 0 to 6, from months 0 to 9, from months 3 to 9, and from months 3 to 12. The median score for the PwD DAD decreased from platform preimplementation at month 3 (median 80.26) to platform postimplementation at month 9 (median 60.53). Please see Table A1 in the Multimedia Appendix 2 for further details.

**Figure 3:**
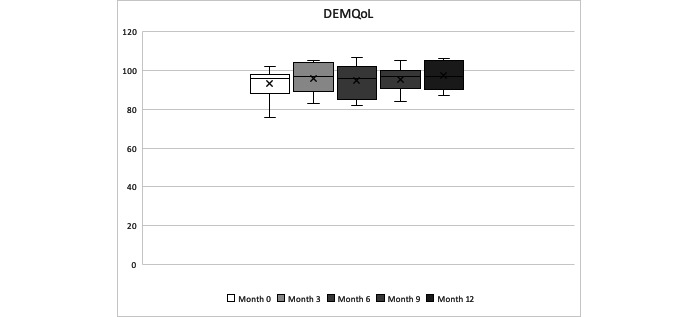
People with dementia self-reported Quality of Life during the year observation period (minimum, quartile 1, median, quartile 3, maximum).

**Figure 4:**
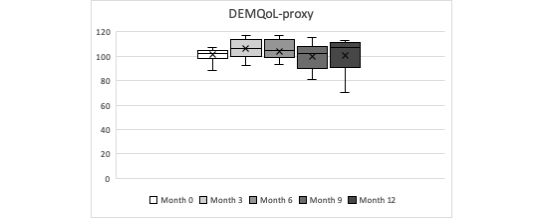
People with dementia informal caregiver reported Quality of Life during the year observation period (minimum, quartile 1, median, quartile 3, maximum).

In terms of the PwD QoL, it was quite stable during the year of follow-up, with no statistically significant change over the year of follow-up time for DEMQoL and DEMQoL-proxy (Friedman test *P*=.78 and *P*=.06, respectively). The overall mean of the DEMQoL-proxy score was 102.26 (SD 10.92), and the overall mean of DEMQoL was 95.21 (SD 7.57), indicating a very good reported QoL from both, the PwD and the caregiver. DEMQoL-proxy scores were, on average, higher than the DEMQoL scores at each time measurement. See Tables 9 and 10 and Figures 3 and 4 for details.

**Table 9:** Dementia Quality of Life score during the year follow-up.

DEMQoL^a^	Month 0	Month 3	Month 6	Month 9	Month 12	Friedman test (*P* value)
Minimum	76	83	82	84	87	.78
Quartile 1	90	91	89	91	91.75	N/A^b^
Median	96	97	96	97	97	N/A
Mean (SD)	93.0 (8.4)	95.7 (7.7)	94.6 (8.2)	95.4 (6.2)	97.4 (7.6)	N/A
Quartile 3	98	103	100	99.7	104.7	N/A
Maximum	102	105	107	105	106	N/A

^a^DEMQoL: Dementia Quality of Life.

^b^N/A: not applicable.

**Table 10:** Dementia Quality of Life-proxy score during the year follow-up.

DEMQoL^a^-proxy	Month 0	Month 3	Month 6	Month 9	Month 12	Friedman test (*P* value)
Minimum	88	92	71	81	70	.06
Quartile 1	99	100.50	101.50	92.50	95.25	N/A^b^
Median	102	106	105	102	107	N/A
Mean (SD)	101.6 (7.4)	106.1 (7.8)	103.5 (12.9)	99.5 (10.9)	100.2 (14.7)	N/A
Quartile 3	104.50	112.50	112.50	105.50	110.50	N/A
Maximum	117	117	117	115	113	N/A

^a^DEMQoL: Dementia Quality of Life.

^b^N/A: not applicable.

#### Informal Caregiver Well-Being Progression

**Figure 5:**
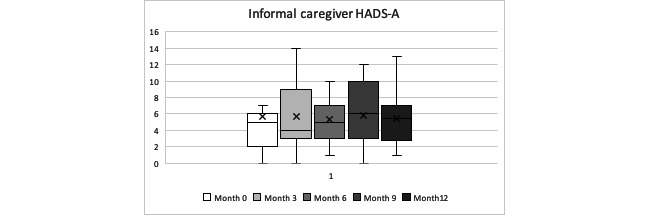
Informal caregivers’ Hospital Anxiety and Depression Scale-A during the year observation period (minimum, quartile 1, median, quartile 3, maximum).

**Figure 6:**
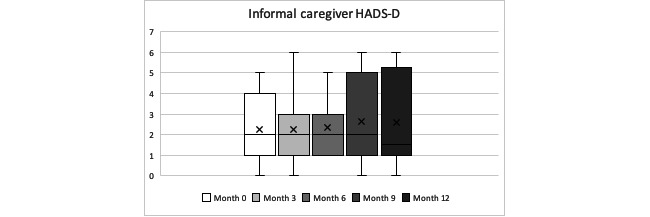
Informal caregivers’ Hospital Anxiety and Depression Scale-D during the year observation period (minimum, quartile 1, median, quartile 3, maximum).

Anxiety and depression scoring did not follow a linear progression, with fluctuations in the scoring along the observation period for both of them. This means that, depending on the assessment month, their anxiety or depression symptoms underwent an improvement or worsening (see individual progression in Multimedia Appendix 3). The overall anxiety mean score (HADS-A) for our informal caregivers was 5.59 (SD 3.91); the global depression mean score (HADS-D) was 2.43 (SD 1.75), with no statistically significant differences between anxiety or depression scores during the year of follow-up (Friedman test *P=.*97 and *P=.*69, respectively). When looking at individual case analysis, 27% (3/11) of caregivers increased their HADS-A score by more than 1.5 points during the year of follow-up, experiencing a worsening of their anxiety, and only 9% (1/11) of caregivers dropped their scores by more than 1.5 points, experiencing an improvement in the anxiety levels. For the HADS-D, 9% (1/11) of caregivers increased their score by more than 1.5 points during the year of follow-up, experiencing a worsening of their depression symptoms, and only 9% (1/11) of caregivers decreased their scores by more than 1.5 points, experiencing an improvement in the depression symptoms. See Tables 11-14 and Figures 5 and 6 for further details.

**Table 11:** Informal caregivers’ anxiety and depression scores during the year observation period.

Hospital Anxiety and Depression Scale-Anxiety	Month 0	Month 3	Month 6	Month 9	Month 12	Friedman test (*P* value)
Minimum	0	0	1	0	1	.97
Quartile 1	3	3	4	3	3.5	N/A^a^
Median	5	4	5	6	6	N/A
Mean (SD)	5.7 (5.4)	5.6 (4.2)	5.2 (2.5)	5.8 (4.0)	5.5 (3.3)	N/A
Quartile 3	6	8.5	7	8	6.7	N/A
Maximum	21	14	10	12	13	N/A

^a^N/A: not applicable.

**Table 12:** Informal caregivers’ anxiety and depression scores during the year observation period.

Hospital Anxiety and Depression Scale-Depression	Month 0	Month 3	Month 6	Month 9	Month 12	Friedman test (*P* value)
Minimum	0	0	1	0	0	.69
Quartile 1	1	1.5	1	1	1	N/A^a^
Median	2	2	2	2	1.50	N/A
Mean (SD)	2.2 (1.6)	2.2 (1.6)	2.3 (1.5)	2.6 (1.9)	2.6 (2.2)	N/A
Quartile 3	4	2.5	3	4	4.50	N/A
Maximum	5	6	5	6	6	N/A

^a^N/A: not applicable.

**Table 13:** Number of caregivers in each Hospital Anxiety and Depression Scale-Anxiety range group at months 0 and 12.

Hospital Anxiety and Depression Scale-Anxiety ranges	Score	Month 0 (n=11), n	Month 12 (n=10), n
Normal	0-7	10	9
Borderline	8-10	0	0
Abnormal (case)	11-21	1	1

**Table 14:** Number of caregivers in each Hospital Anxiety and Depression Scale-Depression range group at months 0 and 12.

Hospital Anxiety and Depression Scale-Depression ranges	Score	Month 0 (n=11), n	Month 12 (n=10), n
Normal	0-7	11	10
Borderline	8-10	0	0
Abnormal (case)	11-21	0	0

**Figure 7:**
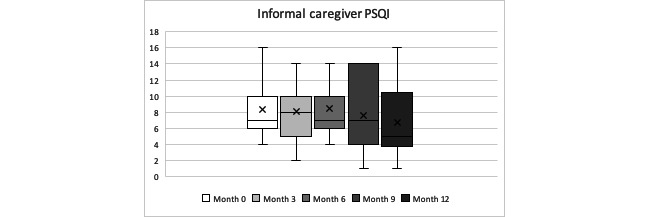
Informal caregivers’ Pittsburgh Sleep Quality Index year progression (minimum, quartile 1, median, quartile 3, maximum).

In terms of sleep quality, our informal caregivers’ overall mean PSQI score was 7.87 (SD 4.01) points. Caregivers’ sleep quality followed a slight progressive decrease in the PSQI of 1.66 points during the year. This means that from the 10 of 11 informal caregivers who had poor sleep quality at month 0, only 5 of 11 had poor sleep quality at month 12. Friedman test indicated that there was a statistically significant difference in caregivers’ sleep quality between each time measurement during the year of follow-up (*P=*.04). When looking at individual cases, it was quite varied and not homogeneous in our participants. See Tables 15-16 and Figure 7 for further details.

**Table 15:** Informal caregivers’ Pittsburgh Sleep Quality Index score during the year progression.

Pittsburgh Sleep Quality Index	Month 0	Month 3	Month 6	Month 9	Month 12	Friedman test (*P* value)
Minimum	4	2	4	1	1	.04
Quartile 1	6.50	5.50	6.50	4.50	4	N/A^a^
Median (SD)	7 (3.4)	8 (3.6)	7 (3.9)	7 (4.8)	5 (4.6)	N/A
Mean	8.36	8.09	8.45	7.64	6.70	N/A
Quartile 3	9.50	10	9.50	12	9.25	N/A
Maximum	16	14	17	14	16	N/A

^a^N/A: not applicable.

**Table 16:** Number of caregivers in each Pittsburgh Sleep Quality Index range group at months 0 and 12.

Pittsburgh Sleep Quality Index ranges	Score	Month 0 (n=11), n	Month 12 (n=10), n
Poor sleep quality	>5	10	5
Normal	<5	1	5

Post hoc pairwise comparisons analysis was conducted using a nonparametric Wilcoxon test to help understand specific differences between the different time intervals within the overall PSQI results, to find where the significance difference relies on between the 5 time point measurements. The Wilcoxon test results revealed a statistical significance in caregivers’ PSQI score from months 3 to 12 and from months 6 to 12. See Table A2 in the Multimedia Appendix 2 for further details.

**Figure 8:**
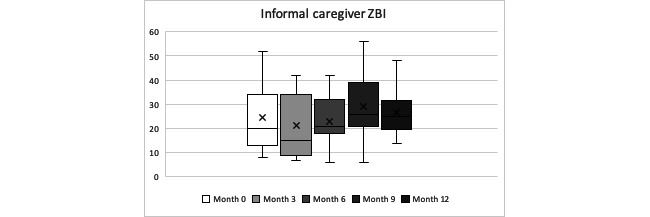
Informal caregivers’ Zarit Burden Interview year progression (minimum, quartile 1, median, quartile 3, maximum).

Overall, informal caregivers’ burden fluctuated during the year observation period. The ZBI mean score over the year was 24.94 (SD 12.55), corresponding to mild-to-moderate levels of burden in our informal caregivers. There was an increase in the proportion of caregivers’ expression of mild-to-moderate levels of burden from an initial 27% (3/11) to 50% (5/11) at the end of the year of follow-up. Friedman test indicated that there was no statistically significant difference in the mean burden score between each time measurement during the year of follow-up (*P=*.13). See Tables 17 and 18 and Figure 8 for further details.

To summarize, we can say that although the global mean for MMSE, DEMQoL, DEMQoL-proxy, HADS-A, HADS-D, and ZBI did not change over time in our PwD and caregiver participants, in some of them, when looking on an individual basis, there were noticeable changes. When individual dyads were analyzed in a case series, we observed a heterogeneous pattern of changes over the year of follow-up. We found 4 cases (Dyad 1, Dyad 3, Dyad 4, and Dyad 10) where there were minimal changes across the full range of measures for the PwD, yet there were changes observed for the caregiver in the cases of D1 and D4. The most common observation was that of a variable pattern of changes where some outcome measures remained stable, and others fluctuated throughout the year, with variation across the PwD and caregiver in each dyad. Detailed individual case analysis descriptions can be found in Multimedia Appendix 1.

**Table 17:** Informal caregivers’ Zarit Burden Interview score during the year progression.

Zarit Burden Interview	Month 0	Month 3	Month 6	Month 9	Month 12	Friedman test (*P* value)
Minimum	8	7	6	6	14	.13
Quartile 1	15	10.5	18.5	21.5	20.2	N/A^a^
Median	20	15	21	26	25	N/A
Mean (SD)	24.5 (14.3)	21.36 (13.0)	23 (10.6)	29.2 (14.4)	26.7 (10.1)	N/A
Quartile 3	29.5	33.5	32	38	28.7	N/A
Maximum	52	42	42	56	48	N/A

^a^N/A: not applicable.

**Table 18:** Number of caregivers in each Zarit Burden Interview range group at months 0 and 12.

Zarit Burden Interview ranges	Score	Month 0 (n=11), n	Month 12 (n=10), n
Little/no burden	0-21	6	4
Mild/moderate	21-40	3	5
Moderate/severe	41-60	2	1
Severe	61-88	0	0

## Discussion

### Principal Findings

The findings from our study suggest that in the majority of measurement outcomes analyzed, there were no significant changes in the PwD or their caregivers’ well-being over the year of follow-up when analyzed as a group. The only instances in which statistically significant changes were observed were a worsening in the functional status of the PwD (using the DAD scale) and a slight improvement in sleep quality for their caregivers (using the PSQI scale). However, in each of these 2 measures, post hoc pairwise comparisons did not indicate any evidence of statistically significant scoring change between the 3 monthly time intervals. This is not unexpected because the small number of participants and the accompanying lack of statistical power limit the statistical inference in this study design. Furthermore, given the observational nature of the study, we cannot attribute that the changes observed were related to the deployment of the platform. However, this lack of statistical significance does not automatically mean that the CHESS platform and its continuous monitoring could offer some advantages in disease progression and disease pattern detection in the long term. When we looked across the individual cases, the results were very varied for each dyad, with no common pattern, but these results support the potential value of individual-level monitoring. As with the group results, we lack evidence to conclude that any of these changes were because of the introduction of the CHESS platform.

The novelty of our work relies on how we approached the PwD and their informal caregivers’ well-being. On the basis of the WHO ICF framework, we have built up a multidimensional profile of the PwD and their informal caregiver to analyze the impact that the health platform could have on their well-being. This could be validated in the future and used as a standard tool to conduct the same analysis with other different s-Health technologies for PwD and their informal caregivers. Furthermore, it is the first time, to our knowledge, that the PwD-caregiver dyad well-being is measured through different outcomes, as usually follow-up studies focus on one single variable measured at different time points [30,31].

Furthermore, as cognition deteriorates, it is more challenging to assess PwD well-being; however, in our study, we used both self-reported and proxy-reported assessments to evaluate how the same situation can be perceived discordantly by the PwD and the caregiver, giving more strength to it. Another strength of our study relies on the time-interval analysis conducted. Measuring the same outcomes, with the same tools, and at a higher frequency during an extended observation period allows us to build up a better outline of a population or an individual and its fluctuation over time. This could provide an opportunity to study some external factors that may influence these variations in time.

### Comparison With Prior Work

Our findings for our PwD–informal caregiver dyads’ well-being progression are in line with similar previous studies in the field, which found that their PwD population did not suffer a significant QoL change during the time they were followed up [30,32]. This is consistent with the literature, which reports that PwD have a progressive adaptation to their cognitive and functional decline, assimilating their limitations and continuing to have positive experiences [33]. The same adaptation is described in the literature for informal caregivers, who do not increase their levels of burden or strain despite the progressive decline of the PwD [34].

Many reviews and meta-analyses have investigated the potential benefits that different types of nonpharmacological interventions can have in the well-being of PwD and their informal caregivers, reducing their levels of burden and depression and improving their QoL, positive affect, physical activity, and self-efficacy, thus having a positive impact on the care recipient [35]. Despite this, they all have something in common: their results do not provide enough evidence to support their use [36]. One of the main reasons that these works argue for that lack of evidence is that there are many different types of studies, including psychoeducational interventions, cognitive behavioral therapy, counseling, support and management, respite, training for the caregiver, or physical health promotion, to name a few. This wide variety of interventions leads to difficulties in comparing the different types of studies [36]. In addition, most of these studies have been found to lack a proper scientific methodology, with different scopes, content, and outcome measures, which decreases their quality and leads them to a lack of evidence [35]. The same issue is noted in the case of technology interventions aimed at improving the PwD’s and their informal caregivers’ QoL. Despite recommending the use of these newly developed technology interventions for improving the well-being of PwD-caregivers at home, the reviews conducted do not provide strong support and claim a lack of evidence in the studies included, arguing having found the same methodology and consistency issues in them [10,37]. Authors in the field claim that there is a need for improvement in the quality of these interventions and that more longitudinal studies need to be conducted to provide evidence of the effect that these interventions can have in the long term [36].

### Limitations

There are some limitations to our study. Despite conducting nonparametric tests, the results cannot be extrapolated to the population because of the small number of participants included. In addition, our PwD sample was quite heterogeneous in terms of the dementia diagnoses and participants’ characteristics. Therefore, our results must be considered in the context of this particular PwD group, their informal caregivers, and their personal living conditions and environment. Our study could also have benefited from a longer follow-up study, as some other studies in the literature indicate.

Another thing to consider is not including the informal caregiver QoL outcome in our study variables when it is considered in the literature as an important factor for assessing the caregiver burden related to continuous care for chronic patients.

We did not differentiate between caregivers who are spouses and those who are children of the PwD. Along the same line, we have not considered the potential impact of the PwD comorbidities in the caregiver, having described only the impact that dementia may have on them.

### Conclusions

The follow-up of this population of PwD and their informal caregivers has shown us that disease progression and their physical and mental well-being do not undergo a significant change during the time, being a more slow and gradual process. The well-being profile created to analyze the potential impact of the CH platform on the PwD–informal caregiver dyad well-being, once validated, could be used as a future tool to conduct the same analyses with other CH technologies for this population.

## Appendix

Multimedia Appendix 1 CHESS platform and components.

Multimedia Appendix 2 Descriptive tables.

Multimedia Appendix 3 Individual dyads description.

## Abbreviations

ADL: activities of daily living
BP: blood pressure
CH: Connected Health
CHESS: Connected HEalth Sustaining home Stay
DAD: Disability Assessment Dementia
DEMQoL: Dementia Quality of Life
HADS: Hospital Anxiety and Depression Scale
HCP: health care professional
ICF: International Classification of Functioning
MMSE: Mini-Mental State Examination
PSQI: Pittsburgh Sleep Quality Index
QoL: quality of life
UCD: University College Dublin
WHO: World Health Organization
ZBI: Zarit Burden Interview
