# Supplementary material for: A Smart Health Platform for Measuring Health and Well-Being Improvement in People With Dementia and Their Informal Caregivers: Usability Study
Source: JMIR Aging. 2020 Jul 23;3(2):e15600. doi: 10.2196/15600 (PMC7413274; doi:10.2196/15600)
Supplement: Multimedia Appendix 1 [file aging_v3i2e15600_app1.docx]

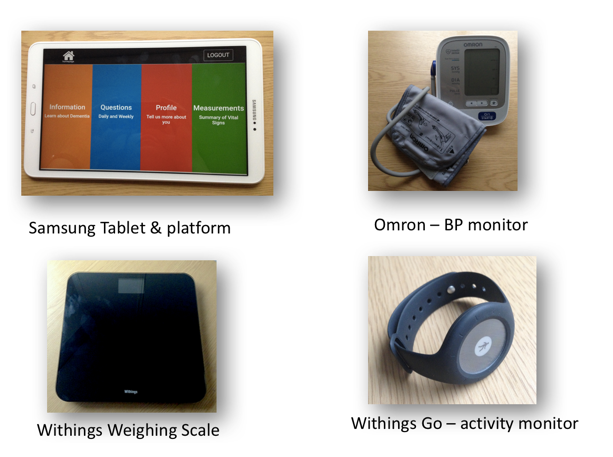


Figure 1. CHESS platform and monitoring devices


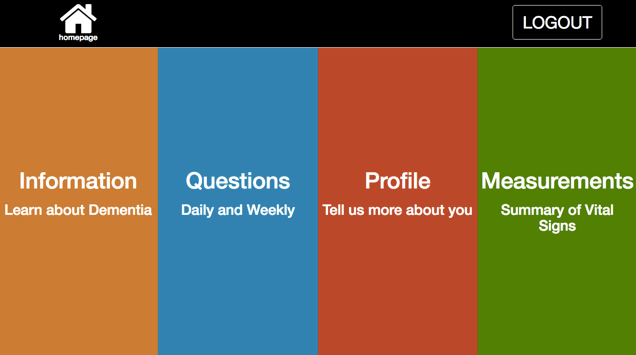


Figure 2. CHESS platform main screen


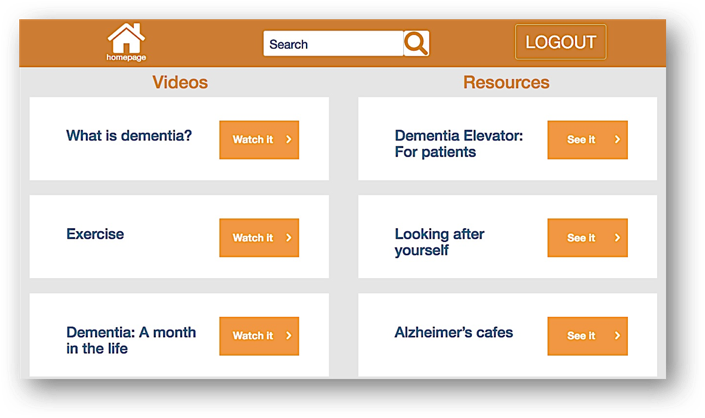


Figure 3. CHESS educational section


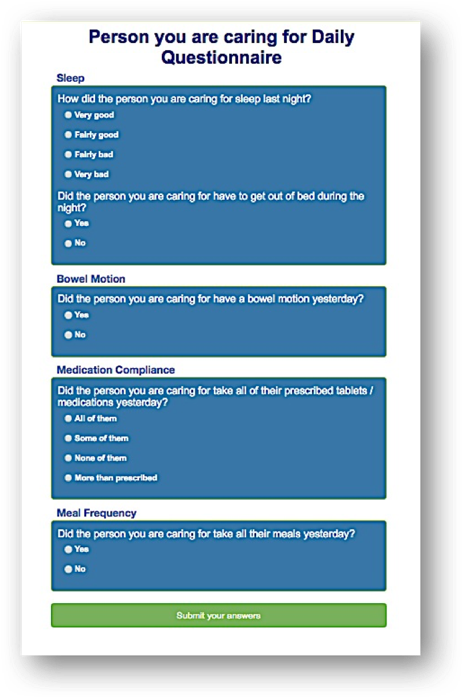


Figure 4. CHESS assessment module


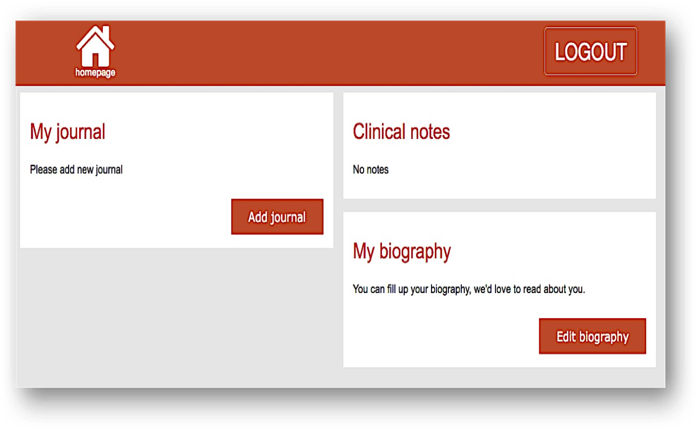


Figure 5. CHESS diary and clinical notes


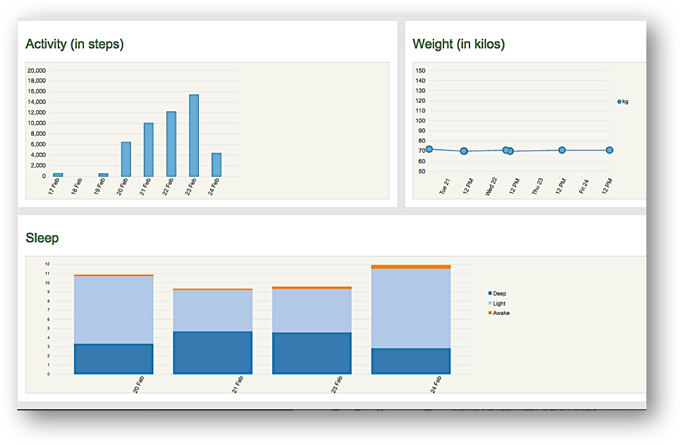


Figure 6. CHESS dashboard
