# Supplementary material for: A Smart Health Platform for Measuring Health and Well-Being Improvement in People With Dementia and Their Informal Caregivers: Usability Study
Source: JMIR Aging. 2020 Jul 23;3(2):e15600. doi: 10.2196/15600 (PMC7413274; doi:10.2196/15600)
Supplement: Multimedia Appendix 2 [file aging_v3i2e15600_app2.docx]

Multimedia appendix 2.

Table A1. Post hoc pairwise comparison results using the non-parametric Wilcoxon test for DAD scores during the year follow up.

| Time measurement | Z | Asymp. Sig (2-tailed)  p value |
| --- | --- | --- |
| **0V3** | -0.20 | 0.83 |
| **0V6** | -2.49 | 0.01 |
| **0V9** | -2.49 | 0.01 |
| **3V6** | -2.31 | 0.02 |
| **3V9** | -2.49 | 0.01 |
| **3V12** | -2.42 | 0.01 |
| **6V9** | -2.29 | 0.02 |
| **6V12** | -1.3 | 0.17 |
| **9V12** | -0.84 | 0.40 |

Table A2. Post hoc pairwise comparison results using the non-parametric Wilcoxon test for PSQI scores during the year follow up.

| Time measurement | Z | Asymp. Sig (2-tailed)  p value |
| --- | --- | --- |
| **0V3** | -0.45 | 0.65 |
| **0V6** | -0.17 | 0.86 |
| **0V9** | -1.13 | 0.25 |
| **3V6** | -0.78 | 0.43 |
| **3V9** | -0.56 | 0.57 |
| **3V12** | -2.03 | 0.04 |
| **6V9** | -1.43 | 0.15 |
| **6V12** | -2.53 | 0.01 |
| **9V12** | -0.34 | 0.73 |
