# Supplementary material for: A Smart Health Platform for Measuring Health and Well-Being Improvement in People With Dementia and Their Informal Caregivers: Usability Study
Source: JMIR Aging. 2020 Jul 23;3(2):e15600. doi: 10.2196/15600 (PMC7413274; doi:10.2196/15600)
Supplement: Multimedia Appendix 3 [file aging_v3i2e15600_app3.docx]

Multimedia appendix 1. Individual dyads case descriptions

##### PwD-informal caregiver dyad 1 (D1)

Pwd-caregiver dyad 1 (D1) experienced an increase in PwD DEMQoL score by 18 points during the year (from 80 points at month-0 to 98 points by month-12). DEMQoL- proxy decreased only by 5 points during the year (from 104 points at month-0 to 99 points at month-12). The informal caregiver PSQI score increased by 3 points during the year (from 13 at month-0 to 16 at month-12), continuing with poor sleep quality. The caregiver ZBI score increased by 16 points during the year (from 13 points at month-0 to 29 points at month-12), changing from suffering ‘little or no burden’ to ‘mild to moderate’ burden. The rest of the outcomes did not experience such a difference during the year follow up. This means that in this dyad case, the PwD maintained an overall high cognitive and functional level during the year follow up although there was a fluctuation in the cognitive levels. Overall QoL was good from the PwD and the informal caregiver perspective. In the caregiver, there is an increase in the burden levels later in the observation period, and a notable sleep interference during the whole year, with low anxiety and depression levels. Please see tables 1 and 2 for further details.

Table 1. D1 PwD outcomes score progression during the year follow up.

| Outcomes variables | Month-0 | Month-3 | Month-6 | Month-9 | Month-12 |
| --- | --- | --- | --- | --- | --- |
| MMSE | 21 | 29 | 27 | 25 | 24 |
| DAD | 97.37 | 100 | 94.29 | 100 | 94.44 |
| DEMQoL | 80 | 89 | 96 | 100 | 98 |
| DEMQOL-P | 104 | 111 | 104 | 95 | 99 |

Table 2. D1 informal caregiver outcomes score progression during the year follow up.

| Outcomes variables | Month-0 | Month-3 | Month-6 | Month-9 | Month-12 |
| --- | --- | --- | --- | --- | --- |
| HADS-A | 6 | 9 | 7 | 6 | 5 |
| HADS-D | 1 | 2 | 3 | 2 | 2 |
| PSQI | 13 | 14 | 17 | 14 | 16 |
| ZBI | 13 | 14 | 20 | 42 | 29 |

##### PwD-informal caregiver dyad 2 (D2)

In PwD-caregiver dyad 2 (D2), the PwD DAD score fluctuated along the year, experiencing an increase in the score by 6.43 points during the year (from 27.78 points at month-0 to 34.21 points by month-12). The caregiver experienced an increase in both HADS-A and HADS-D scoring during the year: HADS-A levels increased by 3 points during the year (from 4 points at month-0 to 7 points at month-12); HADS-D levels increased by 4 points during the year (from 2 points at month-0 to 6 points at month-12). Caregiver PSQI decreased by 2 points during the year (from 6 points at month-0 to 4 points at month-12), moving the caregiver from ‘poor sleep quality’ to ‘normal sleep quality’ range. Caregiver ZBI score suffered many fluctuations during the year, with an overall increase by 7 at the end of the follow up period (from 13 points at month-0 to 20 points at month-12). The rest of the outcomes did not experience such a difference during the year follow up. This means that in this dyad, the PwD maintained a moderate cognitive level during the year follow up, as well as a good QoL from both perspectives, and the functional levels slightly increased by the end of the year. The caregiver was never in the abnormal case for anxiety or depression, and despite the fluctuation in the burden levels, normal levels were maintained at the end. Sleep quality improved during the last months. Please see table 3 and 4 and for further details.

Table 3. D2 PwD outcomes score progression during the year follow up.

| Outcomes variables | Month-0 | Month-3 | Month-6 | Month-9 | Month-12 |
| --- | --- | --- | --- | --- | --- |
| MMSE | 20 | 20 | 17 | 19 | 20 |
| DAD | 27.78 | 37.5 | 30 | 30 | 34.21 |
| DEMQoL | 97 | 85 | 96 | 99 | 94 |
| DEMQOL-P | 103 | 116 | 115 | 115 | 112 |

Table 4. D2 informal caregiver outcomes score progression during the year follow up.

| Outcomes variables | Month-0 | Month-3 | Month-6 | Month-9 | Month-12 |
| --- | --- | --- | --- | --- | --- |
| HADS-A | 4 | 4 | 6 | 4 | 7 |
| HADS-D | 2 | 0 | 2 | 3 | 6 |
| PSQI | 6 | 5 | 5 | 4 | 4 |
| ZBI | 13 | 7 | 22 | 26 | 20 |

##### PwD-informal caregiver dyad 3 (D3)

PwD-caregiver dyad 3 (D3) outcomes scores kept quite stable for both the PwD and the informal caregiver and did not change considerably during the year follow up. Sleep disturbance was the most notable issue for the caregiver. Please see table 5 and 6 for further details.

Table 5. D3 PwD outcomes score progression during the year follow up.

| Outcomes variables | Month-0 | Month-3 | Month-6 | Month-9 | Month-12 |
| --- | --- | --- | --- | --- | --- |
| MMSE | 28 | 30 | 30 | 29 | 30 |
| DAD |  | 95 | 100 | 97.44 | 100 |
| DEMQoL | 98 | 93 | 98 | 91 | 96 |
| DEMQOL-P | 117 | 117 | 117 | 114 | 113 |

Table 6. D3 informal caregiver outcomes score progression during the year follow up.

| Outcomes variables | Month-0 | Month-3 | Month-6 | Month-9 | Month-12 |
| --- | --- | --- | --- | --- | --- |
| HADS-A | 2 | 3 | 3 | 3 | 3 |
| HADS-D | 1 | 2 | 2 | 1 | 1 |
| PSQI | 9 | 10 | 10 | 10 | 10 |
| ZBI | 17 | 9 | 6 | 6 | 14 |

##### PwD-informal caregiver dyad 4 (D4)

In PwD-caregiver dyad 4 (D4) the was a decrease in PwD DAD by 17 points (from 92.11 points at month-0 to 75 points at month-12), and a decrease in PwD QoL (DEMQoL-proxy) by 28 points (from 98 points at month-0 to 70 points at month-12) during the year. The informal caregiver experienced a decrease in PSQI by 4 points (from 16 points at month-0 to 12 points at month-12), maintaining a poor sleep quality. Caregiver ZBI score increased by 28 points along the year (from 20 points at month-0 to 48 points at month-12), moving from a caregiver with ‘little or no burden’ to a caregiver with ‘moderate to severe’ burden. The rest of the outcomes did not experience such a difference during the year follow up. This means that in this dyad, the PwD maintained a good QoL level from the PwD and caregiver perspective and stable cognitive levels during the overall year, but with a worsening in the functional levels. In the case of the caregiver, despite the fluctuations in the anxiety levels, the normal range was maintained at the end, and in this case, the caregiver never got into the depression abnormal case range. The main issues for this caregiver were the constant sleep disturbances and the progressive increase in the burden levels. Please see table 7 and 8 for further details.

Table 7. D4 PwD outcomes score progression during the year follow up.

| Outcomes variables | Month-0 | Month-3 | Month-6 | Month-9 | Month-12 |
| --- | --- | --- | --- | --- | --- |
| MMSE | 30 | 29 | 29 | 28 | 27 |
| DAD | 92.11 | 97.14 | 89.47 | 75 | 75 |
| DEMQoL | 92 | 83 | 82 | 84 | 91 |
| DEMQOL-P | 98 | 92 | 99 | 90 | 70 |

Table 8. D4 informal caregivers outcomes score progression during the year follow up.

| Outcomes variables | Month-0 | Month-3 | Month-6 | Month-9 | Month-12 |
| --- | --- | --- | --- | --- | --- |
| HADS-A | 6 | 8 | 7 | 10 | 7 |
| HADS-D | 1 | 2 | 1 | 2 | 1 |
| PSQI | 16 | 13 | 14 | 14 | 12 |
| ZBI | 20 | 42 | 21 | 37 | 48 |

##### PwD-informal caregiver dyad 5 (D5)

In PwD-caregiver dyad 5 (D5), PwD MMSE score decreased by 7 points (from 19 at month-0 to 12 at month-12) during the year, indicating a cognitive decline from ‘moderate’ to ‘severe’ Dementia. This PwD DAD score decreased in parallel by 18.27 points during the year (moving from 30.77 at month-0 to 12.5 points at month-12). The caregiver PSQI score decreased by 3 points (from 7 points at month-0 to 4 points at month-12) during the year, experiencing improvement from an initial ‘poor sleep quality’ to a ‘normal sleep quality’. ZBI score in the caregiver decreased by 10 points (from 34 points at month-0 to 24 points at month-12) during the year, maintaining the caregiver in a ‘mild to moderate’ burden range. The rest of the outcomes did not experience such a difference during the year follow up. In this dyad, the PwD QoL levels were good and stable during the year, but the cognitive levels decreased at the end to a severe dementia range, as well as the functional levels that suffered an important decrease throughout the year. In the case of the caregiver, the main issue was the burden levels, that were continually in the mild-moderate levels. The sleep quality was an issue at the beginning, but it experienced improvement at the end of the year follow up. Please see table 9 and 10 for further details.

Table 9. D5 PwD outcomes score progression during the year follow up.

| Outcomes variables | Month-0 | Month-3 | Month-6 | Month-9 | Month-12 |
| --- | --- | --- | --- | --- | --- |
| MMSE | 19 | 18 | 16 | 15 | 12 |
| DAD | 30.77 | 20.51 | 15 | 12.51 | 12.51 |
| DEMQoL | 101 | 105 | 107 | 105 | 106 |
| DEMQOL-P | 101 | 104 | 111 | 108 | 106 |

Table 10. D5 informal caregiver outcomes score progression during the year follow up.

| Outcomes variables | Month-0 | Month-3 | Month-6 | Month-9 | Month-12 |
| --- | --- | --- | --- | --- | --- |
| HADS-A | 6 | 4 | 5 | 6 | 5 |
| HADS-D | 4 | 3 | 5 | 5 | 6 |
| PSQI | 7 | 5 | 7 | 5 | 4 |
| ZBI | 34 | 37 | 32 | 37 | 24 |

##### PwD-informal caregiver dyad 6 (D6)

For PwD-caregiver dyad 6 (D6), DAD score in the PwD decreased by 42.18 points (from 97.44 points at month-0 to 55.26 points at month-12) during the year, indicating a high functional decline in the PwD. The rest of the outcomes did not experience such a difference during the year follow up. In this case, the main issue for the PwD was the decrease in the functional levels; and in the case of the caregiver, the sleep disturbances were a constant, as well as the mild-moderate burden levels. Please see table 11 and 12 for further details.

Table 11. D6 PwD outcomes score progression during the year follow up.

| Outcomes variables | Month-0 | Month-3 | Month-6 | Month-9 | Month-12 |
| --- | --- | --- | --- | --- | --- |
| MMSE | 25 | 30 | 24 | 23 | 23 |
| DAD | 97.44 | 94.44 | 87.88 | 75 | 55.26 |
| DEMQoL | 102 | 104 | 102 | 100 | 104 |
| DEMQOL-P | 107 | 114 | 104 | 103 | 109 |

Table 12. D6 informal caregivers outcomes score progression during the year follow up.

| Outcomes variables | Month-0 | Month-3 | Month-6 | Month-9 | Month-12 |
| --- | --- | --- | --- | --- | --- |
| HADS-A | 2 | 1 | 2 | 2 | 1 |
| HADS-D | 2 | 1 | 3 | 1 | 1 |
| PSQI | 7 | 10 | 9 | 7 | 6 |
| ZBI | 22 | 8 | 19 | 22 | 26 |

##### 6.3.2.3.7 PwD-informal caregiver dyad 7 (D7)

In PwD-caregiver dyad 7 (D7), PwD DAD score decreased by 45.92 points (from 68.42 at month-0 to 22.5 points at month-12) during the year. Caregiver HADS-A increased by 2 points (from 4 points at month-0 to 6 points at month-12) during the year, maintaining the ‘normal’ range. Caregiver PSQI decreased by 4 points (from 7 points at month-0 to 3 points at month-12) during the year, which meant an improvement in the caregiver sleep quality from ‘poor’ to ‘normal’. The rest of the outcomes did not experience such a difference during the year follow up. In this dyad, despite the PwD worsening in the functional levels, the caregiver maintained the burden levels in the same mild-moderate range throughout the year and even experienced an improvement in the sleep quality. Please see table 13 and 14 and for further details.

Table 13. D7 PwD outcomes score progression during the year follow up.

| Outcomes variables | Month-0 | Month-3 | Month-6 | Month-9 | Month-12 |
| --- | --- | --- | --- | --- | --- |
| MMSE | 24 | 23 | 23 | 20 | 26 |
| DAD | 68.42 | 47.22 | 36.84 | 25 | 22.51 |
| DEMQoL | 96 | 104 | 85 | 96 | 105 |
| DEMQOL-P | 100 | 106 | 105 | 103 | 111 |

Table 14. D7 PwD informal caregiver score progression during the year follow up.

| Outcomes variables | Month-0 | Month-3 | Month-6 | Month-9 | Month-12 |
| --- | --- | --- | --- | --- | --- |
| HADS-A | 4 | 3 | 5 | 3 | 6 |
| HADS-D | 0 | 2 | 1 | 1 | 1 |
| PSQI | 7 | 8 | 7 | 2 | 3 |
| ZBI | 25 | 24 | 32 | 21 | 28 |

##### PwD-informal caregiver dyad 8 (D8)

In PwD-caregiver dyad 8 (D8), PwD DAD score decreased by 23.68 points (from 73.68 points at month-0 to 50 points at month-12), and a DEMQoL score increase by 11 points (from 76 at month-0 to 87 at month-12) during the year. In the caregiver HADS-A and HADS-D scores decreased during the year: HADS-A decreased by 8 points (from 21 points at month-0 to 13 points at month-12), but sustaining the caregiver in the ‘abnormal’ range; HADS-D decreased by 2 points (from 5 points at month-0 to 3 points at month-12), but the caregiver maintained the ‘normal’ range. Caregiver PSQI score decreased by 6 points (from 10 points at month-0 to 4 points at month-12) during the year, making the caregiver experience an improvement in the sleep quality from ‘poor’ to ‘normal’. Caregiver ZBI score decreased by 9 points (from 48 at month-0 to 39 at month-12) during the year, moving the caregiver burden from ‘moderate to severe’ to ‘mild to moderate’ range. The rest of the outcomes did not experience such a difference during the year follow up. For this dyad, this means that, despite the functional decline experienced in the PwD, the QoL levels are perceived as good from both, the PwD and the informal caregiver perspective. In the case of the caregiver, high anxiety levels are constant during the year, whether burden levels improve progressively as well as sleep quality improves at the end of the follow up. Please see table 15 and 16 for further details.

Table 15. D8 PwD outcomes score progression during the year follow up.

| Outcomes variables | Month-0 | Month-3 | Month-6 | Month-9 | Month-12 |
| --- | --- | --- | --- | --- | --- |
| MMSE | 23 | 18 | 23 | 24 | 20 |
| DAD | 73.68 | 78.38 | 71.79 | 60.53 | 50 |
| DEMQoL | 76 | 93 | 93 | 90 | 87 |
| DEMQOL-P | 88 | 99 | 106 | 86 | 80 |

Table 16. D8 informal caregiver outcomes score progression during the year follow up.

| Outcomes variables | Month-0 | Month-3 | Month-6 | Month-9 | Month-12 |
| --- | --- | --- | --- | --- | --- |
| HADS-A | 21 | 14 | 10 | 12 | 13 |
| HADS-D | 5 | 2 | 2 | 3 | 3 |
| PSQI | 10 | 6 | 6 | 14 | 4 |
| ZBI | 48 | 33 | 32 | 39 | 39 |

##### PwD-informal caregiver dyad 9 (D9)

In PwD-caregiver dyad 9 (D9) the PwD DAD, that decreased by 9.34 points (from 86.84 points at month-0 to 77.5 points at month-12) during the year. The PwD cognitive levels decreased by 3 points (from 23 points at month-0 to 20 points at month-12), indicating that the PwD suffered a cognitive decline from ‘mild’ to ‘moderate’ level. The rest of the outcomes did not experience such a difference during the year follow up. The main issues here were the PwD cognitive and functional decline, as well as the caregiver sleep disturbances that were persistent during the year follow up. Please see table 17 and 18 for further details.

Table 17. D9 PwD outcomes score progression during the year follow up.

| Outcomes variables | Month-0 | Month-3 | Month-6 | Month-9 | Month-12 |
| --- | --- | --- | --- | --- | --- |
| MMSE | 23 | 23 | 21 | 19 | 20 |
| DAD | 86.84 | 89.47 | 62.51 | 60 | 77.52 |
| DEMQoL | 88 | 98 | 83 | 91 | 87 |
| DEMQOL-P | 105 | 108 | 114 | 98 | 108 |

Table 18. D9 PwD informal caregiver score progression during the year follow up.

| Outcomes variables | Month-0 | Month-3 | Month-6 | Month-9 | Month-12 |
| --- | --- | --- | --- | --- | --- |
| HADS-A | 7 | 6 | 5 | 6 | 6 |
| HADS-D | 4 | 6 | 5 | 6 | 5 |
| PSQI | 6 | 10 | 7 | 5 | 7 |
| ZBI | 18 | 15 | 18 | 24 | 21 |

##### PwD-informal caregiver dyad 10 (D10)

The case of PwD-caregiver dyad 10 (D10) was quite similar to the previous dyad, with very balanced scores during the year progression. The only significant change in the PwD was the DAD scores, which decreased by 17.37 points (from 97.37 points at month-0 to 80 points at month-12) during the year. In the caregiver, HADS-A experienced an increase by 2 points (from 0 points at month-0 to 2 points at month-12) during the year but maintaining the ‘normal’ range for anxiety. The rest of the outcomes did not experience such a difference during the year follow up. The main issue in this dyad was the PwD functional levels decline. Please see table 19 and 20 for further details.

Table 19. D10 PwD outcomes scores progression during the year follow up.

| Outcomes variables | Month-0 | Month-3 | Month-6 | Month-9 | Month-12 |
| --- | --- | --- | --- | --- | --- |
| MMSE | 28 | 27 | 28 | 24 | 26 |
| DAD | 97.37 | 80 | 86.84 | 85 | 80 |
| DEMQoL | 98 | 97 | 103 | 98 | 106 |
| DEMQOL-P | 102 | 100 | 93 | 102 | 94 |

Table 20. D10 informal caregiver outcomes scores progression during the year follow up.

| Outcomes variables | Month-0 | Month-3 | Month-6 | Month-9 | Month-12 |
| --- | --- | --- | --- | --- | --- |
| HADS-A | 0 | 0 | 1 | 0 | 2 |
| HADS-D | 1 | 1 | 1 | 0 | 0 |
| PSQI | 4 | 2 | 4 | 1 | 1 |
| ZBI | 8 | 12 | 9 | 12 | 18 |

##### PwD-informal caregiver dyad 11 (D11)

In the case of PwD-caregiver dyad 11 (D11), things were very different since this was the dyad that suffered more changes and decline during the year progression. Month-12 visit data is missing due to the PwD deep functional and cognitive decline, that did not allow to complete the questionnaires either with him or the caregiver, who was having a hard time answering what could be considered sensitive questions about themselves. In the PwD, the MMSE score could not be obtained at month-0, but experienced a decrease by 7 points (from 18 at month-3 to 11 at month-9) during the follow up, implying a PwD cognitive decline from ‘moderate’ to ‘severe’ Dementia range. The PwD DAD score decreased by 21.09 points (from 43.59 points at month-0 to 22.50 points at month-9), and the PwD DEMQoL-p decreased by 12 points (from 93 points at month-3 to 81 points at month-12) during the follow up. Caregiver HADS-A score increased by 7 points (from 5 points at month-0 to 12 points at month-9) during the follow up, changing from a ‘normal case’ to an ‘abnormal case’. This indicates that the progressive and quick functional and cognitive decline experienced by the PwD was translated into an increase in the caregiver levels of anxiety, maintaining quite balanced HADS-D scoring levels during the follow up. The rest of the outcomes did not experience such a difference during the year follow up. Please see table 21 and 22 for further details.

Table 21. D11 PwD outcomes score progression during the year follow up.

| Outcomes variables | Month-0 | Month-3 | Month-6 | Month-9 | Month-12 |
| --- | --- | --- | --- | --- | --- |
| MMSE |  | 18 | 11 |  |  |
| DAD | 43.59 | 53.85 | 42.11 | 22.51 |  |
| DEMQoL | 96 | 102 | 96 |  |  |
| DEMQOL-P | 93 | 101 | 71 | 81 |  |

Table 22. D11 informal caregiver outcomes score progression during the year follow up.

| Outcomes variables | Month-0 | Month-3 | Month-6 | Month-9 | Month-12 |
| --- | --- | --- | --- | --- | --- |
| HADS-A | 5 | 10 | 7 | 12 |  |
| HADS-D | 4 | 4 | 1 | 5 |  |
| PSQI | 7 | 6 | 7 | 8 |  |
| ZBI | 52 | 34 | 42 | 56 |  |
